# Survey of transcripts expressed by the invasive juvenile stage of the liver fluke *Fasciola hepatica*

**DOI:** 10.1186/1471-2164-11-227

**Published:** 2010-04-07

**Authors:** Martín Cancela, Natalia Ruétalo, Nicolás Dell'Oca, Edileuza da Silva, Pablo Smircich, Gabriel Rinaldi, Leda Roche, Carlos Carmona, Fernando Alvarez-Valín, Arnaldo Zaha, José F Tort

**Affiliations:** 1 Departamento de Biologia Molecular e Biotecnologia, Instituto de Biociências e Programa de Pós-Graduação em Biologia Celular e Molecular, Centro de Biotecnologia, Universidade Federal do Rio Grande do Sul, UFRGS, Porto Alegre, Brazil; 2 Departamento de Genética, Facultad de Medicina, Universidad de la República, UDELAR, Montevideo, Uruguay; 3 Laboratorio de Biomatemáticas, Instituto de Biología, Facultad de Ciencias, Universidad de la República, UDELAR, Montevideo, Uruguay; 4 Unidad de Biología Parasitaria, Instituto de Biología, Facultad de Ciencias, Universidad de la República, UDELAR, Montevideo, Uruguay

## Abstract

**Background:**

The common liver fluke *Fasciola hepatica *is the agent of a zoonosis with significant economic consequences in livestock production worldwide, and increasing relevance to human health in developing countries. Although flukicidal drugs are available, re-infection and emerging resistance are demanding new efficient and inexpensive control strategies. Understanding the molecular mechanisms underlying the host-parasite interaction provide relevant clues in this search, while enlightening the physiological adaptations to parasitism. Genomics and transcriptomics are still in their infancy in *F. hepatica*, with very scarce information available from the invasive newly excysted juveniles (NEJ). Here we provide an initial glimpse to the transcriptomics of the NEJ, the first stage to interact with the mammalian host.

**Results:**

We catalogued more than 500 clusters generated from the analysis of *F. hepatica *juvenile expressed sequence tags (EST), several of them not detected in the adult stage. A set of putative *F. hepatica *specific transcripts, and a group of sequences conserved exclusively in flatworms were identified. These novel sequences along with a set of parasite transcripts absent in the host genomes are putative new targets for future anti-parasitic drugs or vaccine development.

Comparisons of the *F. hepatica *sequences with other metazoans genomes or EST databases were consistent with the basal positioning of flatworms in the bilaterian phylogeny. Notably, GC content, codon usage and amino acid frequencies are remarkably different in Schistosomes to *F. hepatica *and other trematodes.

Functional annotation of predicted proteins showed a general representation of diverse biological functions. Besides proteases and antioxidant enzymes expected to participate in the early interaction with the host, various proteins involved in gene expression, protein synthesis, cell signaling and mitochondrial enzymes were identified. Differential expression of secreted protease gene family members between juvenile and adult stages may respond to different needs during host colonization.

**Conclusion:**

The knowledge of the genes expressed by the invasive stage of *Fasciola hepatica *is a starting point to unravel key aspects of this parasite's biology. The integration of the emerging transcriptomics, and proteomics data and the advent of functional genomics tools in this organism are positioning *F. hepatica *as an interesting model for trematode biology.

## Background

*Fasciola hepatica*, the common liver fluke, is recognized as one of the most important parasitic helminths affecting livestock worldwide. Along with the related species *F. gigantica*, *F. hepatica *is responsible for massive economic losses estimated globally at 3.2 bn USD mainly due to reduction in meat, wool and milk output in infected animals, with additional costs derived from liver condemnation and flukicide drugs [1]. During the last decade, its relevance as a zoonotic agent in parts of Latin America and Africa has also emerged, with millions at risk of infection [2,3]. Although effective drugs such as triclabendazole are available, they only provide interim control of the disease, since cattle and sheep are easily reinfected. Moreover, drug resistance against triclabendazole has emerged in Australia and European countries (Ireland, The Netherlands, U.K. and Spain) jeopardizing the long term sustainability of this control strategy [4].

The life cycle of *F. hepatica *is complex and includes a snail and a mammal as intermediate and definitive hosts respectively. Mammals get infected by ingestion of the quiescent larvae (metacercariae) encysted in the vegetation. An interplay of extrinsic signals from the host (digestive enzymes, bile salts, redox potential, pH, temperature among others) and intrinsic factors from the parasite (enzymes and secretions) determine the emergence of a motile larvae [5]. The newly excysted juveniles (NEJ) actively penetrate and transverse the gut wall into the peritoneal cavity within two or three hours. By four or five days post-infection the parasites reach and penetrate the liver, and continue burrowing through the parenchyma for several weeks. Within the major bile ducts the parasites mature and start to release eggs, that can be found in the bile and feces from 8 weeks post-infection [6].

Unlike mature flukes living in the immunologically safe environment of the bile ducts, NEJ are susceptible targets of the immune response. Only 5-10% of the inoculum in cattle, and 20-25% in sheep reach maturity in experimental infections, indicating that a great part of the emerged juveniles either fail entering the gut or are killed during the migrating phase [7,8]. Vaccination studies also show that effective protection is correlated with reduced liver damage, a signature of previous destruction of the early NEJs. Despite the crucial role of this stage in determining the further success of the infective process, information regarding NEJs, is very limited, mainly due to the scarce availability of material to explore diverse aspects of the parasite biology. Principal roles for stage specific proteases and antioxidant enzymes in the early infection have been demonstrated by us and others [9-12]. Recent proteomic studies were able to reveal important differences among *F. hepatica *stages [13-15]. However, the identification of the juvenile specific proteins was limited by the paucity of mRNA sequences to match to peptide mass fingerprinting data. While more than 200 protein sequences and 10,000 EST are available from the adult stage, only 22 mRNA sequences from NEJ (mainly corresponding to cathepsin B and L-like cysteine proteinases) were deposited at the Genbank by July 2009. Consequently we decided to conduct a transcriptomic analysis in order to identify the gene repertoire expressed by the invasive stage of *F. hepatica*. Transcriptomic approaches in *Schistosoma mansoni *and *S. japonicum *have provided a thorough coverage of the genes expressed by diverse stages [16,17]. Furthermore, they have been invaluable tools for the assembly and annotation of the recently released genomes of these important human parasites [18,19], opening new avenues for discovery [20,21]. EST have also been applied successfully to a limited set of other trematodes, namely *Echinostoma paraensei *[22], *Clonorchis sinensis *[23-25], *Paragonimus westermani *[26] and *Opisthorchis viverrini *[27].

Here we report the analysis of a limited set of NEJ expressed sequence tags, identifying putative stage, species and flatworm specific sequences. This first glimpse of the physiology of the invasive larvae opens new prospects for the understanding of the host-parasite interaction eventually leading to the development of new mechanisms to control fasciolosis, and warrants further analysis using new generation sequencing technologies.

## Results and Discussion

### Construction of a newly excysted juvenile *F. hepatica *cDNA library

In order to identify the genes expressed during the invasion process of the platyhelminth *F. hepatica*, we constructed a full length enriched cDNA library using a modified protocol based on selective amplification of capped polyadenylated RNA species. Since the starting parasite material was limiting, a modified size fractioning step of the products was introduced in order to improve the yield [28] (Additional File 1). More than four thousand reads were produced and analyzed using the Partigene pipeline [29]. Quality and vector trimming drastically reduced the starting 4319 ESTs to 1684 high quality sequences, mainly due to the presence of multimers of the adapters used in the generation of the libraries (see methods). This setback could be expected considering the minimal amount of starting material, and might be corrected using 5' blocked adapters in lower concentrations.

The resulting high quality sequences were clustered into 517 different contigs (249 clusters and 268 singletons), 74.6% of them showing significant similarity (*E value *< 1e^-05^) with protein coding genes deposited in public sequence databases, indicating a good representation of cDNAs in this library (Table 1). The most highly abundant EST in juvenile *F. hepatica *(13.5% of total reads) corresponds to the large subunit of the mitochondrial ribosomal RNA (LSU rRNA), and was discarded from further analysis. Polyadenylated LSU rRNA has already been described in other platyhelminths [28], and in fact, *F. hepatica *LSU rRNA has been reported to represent about 10% of the adult transcripts [30]. Considering that only 22 sequences from NEJ were available in Genbank by July 2009 (15 of them encoding cathepsins), the present report represents a pertinent contribution to the knowledge of the genes expressed by the invasive stage of the common liver fluke.

**Table 1:** Overview of *F. hepatica *NEJ ESTs assembly

NEJ LIBRARY	Partigene
EST generated	4319
Submissable EST	1684
Contigs	516
Clusters	248
Singletons	268
Mitochondrial LSU RNA	228
Contigs with Blast hits	386
Contigs with GO assignments	174
Contigs with Pfam hits	179
Average insert size	347

### Comparison and validation of the FhNEJ ESTs with other databases

In order to establish if the obtained contig sequences correspond to validated transcripts, we compared them to different available databases, including ESTs from the adult *F. hepatica *stage, predicted coding sequences from selected organisms with complete genomes, and transcriptomes of other eukaryotes representing the main lineages in the metazoan diversity (Additional File 2). To compare the data obtained from the juvenile stage to the adult sequences, we retrieved and analyzed using the Partigene pipeline more than 10,000 EST reads from *F. hepatica *adult worms available at the Wellcome Trust Sanger Institute, obtaining 4089 contigs (1879 clusters and 2210 singletons), 58% of them showing significant blast hits (*E value *< 1e^-05^) with publicly available databases (Additional File 3). These results are very similar to a recently reported analysis of the same dataset performed using a different pipeline [13].

**Figure 1:**
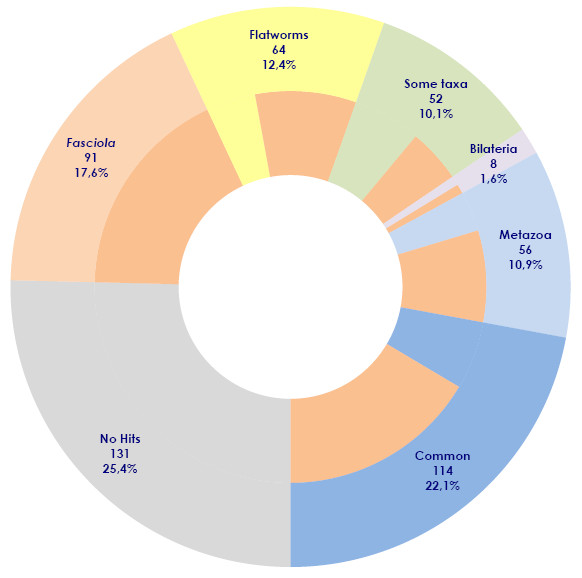
**Distribution of hits of *F. hepatica *NEJ contigs against diverse databases**. The NEJ contigs generated by the Partigene pipeline were compared by Blast with the set of databases corresponding to diverse taxons (listed in Additional file 2), and the results aggregated by their conservation in diverse taxonomic groups. Groups correspond to sequences finding hits in all taxa tested (common), all metazoans, all bilaterians or exclusively in flatworms or in *F. hepatica *adult stage ESTs. Sequences producing positive hits with some taxa but not with others (i.e. absent in deuterostomes) are also indicated, and sequences producing no hits are depicted in grey. Sequences producing hits with the adult stage ESTs dataset within each category are indicated in orange in the inner circle.

More than half of the juvenile contigs (55.3%) were also found in adult ESTs (Figure 1). A set of 91 juvenile contigs (17.6%), also present in adults, showed no homology to sequences in other databases, suggesting that they might represent *Fasciola *specific transcripts expressed in diverse stages of the parasite life cycle. On the other hand, there are several juvenile contigs that are absent from the adult database, although represented in other organisms suggesting that they might represent stage specific transcripts (Figure 1). A set of 114 juvenile contigs (22,1%) were common to all other organisms searched indicating core eukaryotic functions such as ribosomal proteins and common enzymes. The absence of some of them from the adult dataset might suggest that the representation of the adult libraries is still partial. Interestingly, 64 contigs (12.4%) are shared only within flatworms, corresponding to conserved uncharacterized transcripts that might be relevant to parasitism. Also 56 contigs (10.9%) are shared only within metazoans and absent in the non metazoan choanoflagellate *Monosiga brevicollis*, suggesting that they represent metazoan innovations.

**Figure 2:**
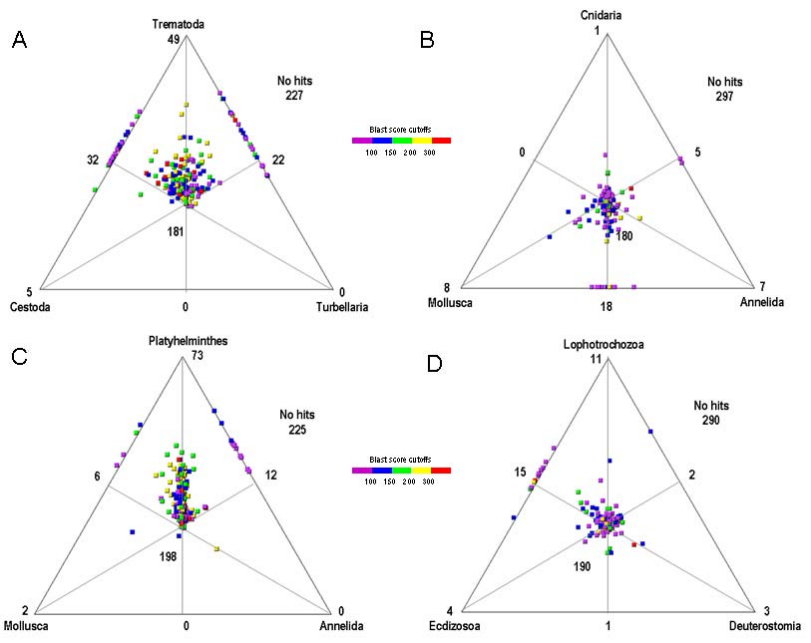
**Three way comparisons of *F. hepatica *juvenile contigs**. The complete set of contigs generated by the Partigene pipeline was compared by tblastx with a set of ESTs or cDNA databases indicated in Additional file 2. The resulting tblastx scores were transformed in coordinates and represented in a triangular graph with Simitri. Each sequence is represented by a dot colour coded by its aggregated blast score and placed in the middle area if found in the three databases, on the corresponding cathetus if found in two databases. Sequences that match in only one of the databases or have no hits are not depicted, but their numbers are indicated. For clarity angle bisector lines were added. Comparisons are shown among **(A) **trematodes, cestodes, and turbellaria **(B) **cnidarians, mollusks, and annelids **(C) **flatworms (excluding *F. hepatica*), mollusks, and annelids **(D) **lophotrochozoans (excluding platyhelminths) ecdysozoans, and deuterostomes.

To further characterize conservation patterns between different metazoan lineages, we analyzed the distribution of tblastx hits by three-way comparisons using the Simitri program [31]. As expected, the *F. hepatica *predicted genes are more similar to homologues from other trematodes rather than cestodes and turbellaria, and to all flatworms rather than other protostomes, supporting the monophylectic origin of flatworms (Figure 2A, C). Consistent with the reports from the schistosomes genomes, we detected slightly more shared genes (being them also more similar) with the complete genomes of vertebrates than with insects and nematodes [18,19]. These results further support the idea that ancient genomes were gene rich, and that lineage specific gene gain and loss events were frequent during metazoan evolution, particularly within the ecdysozoans [32]. While the relevance of genes shared between trematodes and their hosts has been highlighted, since they may be crucial for parasite adaptation to the host [33], the inverse situation (genes present in the parasite but absent in their hosts), might provide relevant candidates for anti-parasitic intervention.

Additionally, since we included in the study partial genomes from other lophotrochozoans (annelids and mollusks) we were able to compare the *Fasciola *dataset to these organisms and other phyla. This is relevant since flatworm position in modern phylogeny is still debated, being placed either within or as sister group of the lophotrochozoa [34-36]. The conserved set of liver fluke genes is almost equally distant from cnidarians, mollusks, and annelids, but slightly closer to the two lophotrochozoans than the model ecdysozoans or vertebrates (Figure 2B, D, and Additional File 4). The trend in this (and all other comparisons performed) were maintained when including the 4089 *F. hepatica *adult contigs suggesting that the effects observed might not be due to sampling bias (data not shown). The comparisons here presented are consistent with the placement of flatworms basal to the lophotrochozoans.

### Compositional characteristics of *F. hepatica *predicted proteins

**Figure 3:**
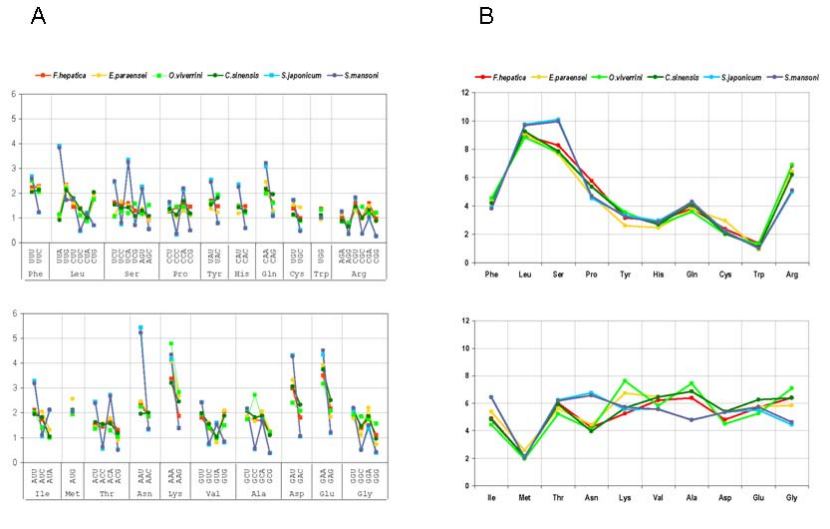
**Codon and amino acid usage in different trematodes**. Coding sequences of more than 500 amino acids from diverse trematodes were collected and analyzed for their codon and amino acid usage. The graphs indicate **(A) **the total frequency of use of each codon in diverse trematodes, **(B) **the total frequency of use of each amino acid in the diverse trematodes species analyzed: *Fasciola hepatica *(red) *Echinostoma paraensei *(yellow) *Opisthorchis viverrini *(green) *Clonorchis sinensis *(purple), *Schistosoma japonicum *(sky-blue) and *Schistosoma mansoni *(blue).

The average G+C content of the *F. hepatica *ESTs (both juvenile or adult) was 45%, a value substantially higher than in *S. mansoni *and *S. japonicum *(34%) [37]. Since variation in GC content can result in skewed codon usage [38], we analyzed the frequency of codons and amino acids of the predicted protein coding sequences in all *F. hepatica *available assemble ESTs (NEJ and adult stage), and compared it to those observed in other trematodes. As indicated in Figure 3A, there is a detectable difference in codon frequency, between the schistosomes and the other trematodes (including *F. hepatica*). Schistosomes prefer the most AU rich codon of each synonymous family, and are also strongly biased against C or G in the third codon position confirming early predictions obtained with limited gene sets [39]. More striking is the fact that significant differences were also found at the amino acid level, where schistosomes uses less Arg, Ala and Gly, and are enriched in Asn, Ile and Ser (Figure 3B). In a recent paper the tRNA complement of *S. mansoni *and *S. japonicum *is analyzed, but no significant correlation between tRNA copy number with the overall codon usage were found in any of the species [40]. The biological and evolutionary significance of the differences here observed is not clear, and deserves further consideration. In any case, these results raise the question that schistosomes might represent a more divergent than expected model for other trematodes.

### Gene Ontology classification and functional annotation

**Figure 4:**
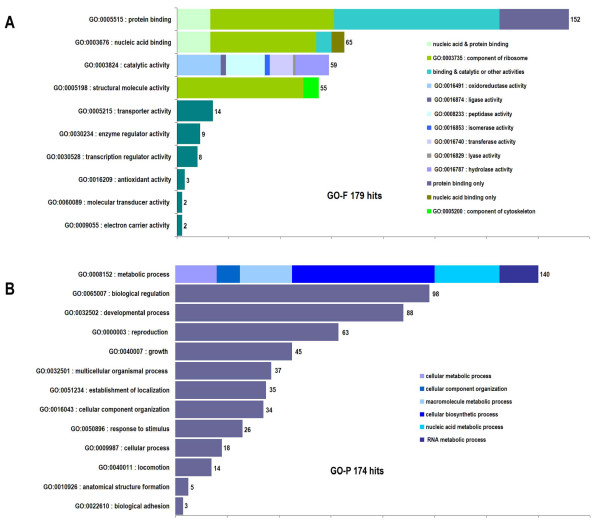
**Assignation of the juvenile *F. hepatica *contigs to the major categories of Gene Ontology**. The NEJ contigs generated by the Partigene pipeline were compared with Gene Ontology, and the ontologies recovered mapped to the upper category using an in-house modified version of go-slim. Note that each contig might map to more than one category within each ontology **(A) **Molecular function assignation: the more abundant categories (binding, catalytic and structural component were further subdivided) **(B) **Cellular component assignation, as in (A) the metabolic process category was subdivided in its child categories.

Gene Ontology (GO) provides a useful way of classifying and annotating sequence information. Our analysis of the *F. hepatica *juvenile dataset showed up to 179 NEJ contigs with GO assignment. The molecular function classification showed a predominance of the binding category overlapping with almost all other categorizations, followed by enzymes (catalytic activity) and structural components. The discrimination within the binding class showed three main divisions of similar relevance, two overlapping with enzymes and ribosomal proteins and a set identified as protein and DNA binding associated with regulatory functions (Figure 4A). The more represented biological process categories were linked with metabolism, regulation and development (Figure 4B), showing a consistent assignment of GO cellular components (data not shown). Functional annotation of predicted proteins showed a general representation of the diverse biological functions. Proteases and antioxidant enzymes should be highlighted since they have long been under scrutiny for their putative involvement in invasion and immune evasion processes [9,10,41-49]. Novel proteins included ribosomal proteins (Additional File 5) several factors associated with protein and gene expression, cell signaling and apoptosis, as well as orthologues of candidate antigens that induce protection against other helminthiasis. They include tetraspanin-like protein [50], a membrane spanning protein located at the tegument of *S. mansoni*, Sm22.6 tegument antigen [51], and venom allergen-like (VAL) proteins, a candidate vaccine antigen against *Necator americanus *and *Ancylostoma caninum *[52-55].

### Relevant molecules for parasitism

Despite the small size of our juvenile library the more represented sequences included proteinases and antioxidant enzymes previously reported as being predominantly expressed in NEJ [12,56-60], together with predicted proteins of unknown function conserved only in *F. hepatica *or in other trematodes but not in other taxa (Table 2).

**Table 2:** Contigs including more reads in the *F. hepatica *NEJ ESTs assembly

Contig	Length	Reads	Signal_P*	TMHMM**	Description***	Distribution							
						*FHE*	*TRE*	*CES*	*TUR*	*CND*	*LTZ*	*ECZ*	*DTS*
FHC00023	824	57	SPep	0	Similar to FHA01510_1	**X**							
FHC00067	483	30	SPep	1	No hit								
FHC00049	1130	27	-	0	cathepsin B3	**X**	X	X	X	X	X	X	X
FHC00068	615	21	-	0	Similar to AT006824 *C. sinensis *clone	X	x						
FHC00138	876	18	-	0	Thioredoxin peroxidise	X	**X**	X	X	X	X	X	X
FHC00005	792	16	SAnc	3	Similar to AT009818 *C. sinensis *clone	X	x						
FHC00024	1064	14	SPep	0	cathepsin L3	**X**	X	X	X	X	X	X	X
FHC00014	612	12	SAnc	2	Similar to AT009818 *C. sinensis *clone	**X**	x						
FHC00006	501	12	SPep	1	Similar to AT009816 *C. sinensis *clone	**X**	x						
FHC00095	699	11	-	0	Similar to FhAE00302	**X**							
FHC00061	819	10	SAnc	3	Similar to AT008757 *C. sinensis *clone	**X**	x						
FHC00091	443	10	-	0	cysteine-rich intestinal protein	**X**	x	x	x	x	x	x	x
FHC00340	362	9	-	0	Similar to FHA01532_1	X							
FHC00174	413	9	-	0	Similar to OvAE2228 *O. viverrini*		X	x	x	x	x		
FHC00054	610	8	-	0	pro-cathepsin B1	X	X	X	X	X	X	X	X
FHC00376	473	8	SAnc	1	FN5 protein	X	x	x	x		x		x
FHC00101	564	7	-	0	Peptidyl-prolyl cis-trans isomerase	**X**	X	X	X	X	X	X	X
FHC00017	1131	7	SPep	0	pro-cathepsin B2	**X**	**X**	X	X	**X**	**X**	X	X
FHC00175	930	7	-	0	Ribosomal protein S2	**X**	**X**	X	X	X	X	**X**	X
FHC00013	252	7	-	0	Ribosomal protein S29	X	x	x	x	x	x	x	x
FHC00187	282	7	-	0	Calcium-binding protein	X	x	x	x	x	x		
FHC00030	680	6	SPep	0	Cellular nucleic acid-binding protein	X	x	x	x	x	x	x	x
FHC00018	289	6	-	0	Similar to FhAE00481	X	x						
FHC00038	416	6	SAnc	1	no hit								
FHC00172	476	6	SPep	0	Similar to FhAE00307	X				x			

* Signal P results indicating prediction of a signal peptide or a signal anchor

** Number of predicted transmembrane domains as predicted by THMMM is indicated

*** Presence of a putatively relevant blast hit in different taxons is indicated. Hits below e^-10 ^represented by a lowercase x, under e^-50 ^by uppercase X, and e^-100 ^in bold X.

Columns headings are FHE (F. hepatica adult stage), TRE (other trematoda), CES (cestoda), TUR (turbellaria), CND (cnidaria), LTZ (lophotrochozoa), ECZ (ecdysozoa), DTS (deuterostomia)

Secreted cathepsins were among the more represented transcripts in juvenile ESTs, and also in the adult dataset (Table 2, Additional File 6). A more detailed analysis of these transcripts showed that different isoforms are are being expressed by the invading and adult stage. While cathepsins L3, L4 and L6 are detected in the juvenile ESTs, they are absent from the much larger adult dataset (with the exception of cathepsins L4). Proteomic analysis have shown that cathepsins L1 and L2 are clearly predominant in adults, in agreement with the relative abundance of their transcripts in the adult EST database [13] (Additional File 6), and it has been proposed that the repertoire of cathepsin Ls gradually change from those expressed in juveniles to a different set characteristic of the adults worms [13-15]. Interestingly, it has recently been reported that the juvenile predominant cathepsin L3 has a strong collagenase activity, that might result essential for the invasion process [61], while the "adult" cathepsin L1 is involved in hemoglobin degradation [14].

We found evidence that within the less characterized cathepsin B gene family a similar phenomenon might be taking place. The cathepsin B forms that appear as frequent in juveniles are quite distinct to the cathepsin B transcripts found in the adult stage dataset (Additional Files 7 and 8), suggesting that they might also be functionally distinct; cathepsin B1 functions as a digestive enzyme in the juvenile gut [62].

Further evidence that changing repertoires of enzymes within gene families might be a common theme in the parasite adaptation to the diverse environments found in their hosts is provided by the legumains. These enzymes have been proposed to have a relevant role activating other enzymes in helminth proteolytic cascades [12,13,63-67]. A novel legumain detected in the juvenile ESTs, legumain 3 has an inverted expression pattern with the previously reported legumain isolated from adult worms (Additional File 9, panels A, B). Besides the already described cathepsins and legumains, the degradome of the juvenile liver fluke was enriched by other proteases, including a novel serine proteinase, calcium-dependent cysteine proteinases (calpains), and components of the proteasome and ubiquitin pathway (Table 3). Proteinase inhibitors like cystatins were also produced by the juvenile larvae. These might modulate parasite proteases on the host immune response as was described for nematode cystatins [68-71].

**Table 3:** Putative host interacting proteins of NEJ of *Fasciola hepatica*

Contig	e value	Best Hit Accesion	Species	Description	Pfam ID
**Proteases**					
FHC00852	4,00E-68	Smp173840|29601	*S. mansoni*	26S protease regulatory subunit	PF00004.21
FHC00017	1,00E-172	CAD32937	*F. hepatica*	Pro-cathepsin B2	PF00112.15
FHC00024	0	ACM67632	*F. hepatica*	Cathepsin 2L	PF00112.15
FHC00049	0	ABU62925	*F. hepatica*	Cathepsin B3	PF00112.15
FHC00054	1,00E-103	CAD32937	*F. hepatica*	Pro-cathepsin B2	PF00112.15
FHC00092	2,00E-57	ABU62925	*F. hepatica*	Cathepsin B	PF00112.15
FHC00154	3,00E-40	ABZ80402	*F. hepatica*	Cathepsin L6	PF00112.15
FHC00522	1,00E-34	ABW75768	*F. hepatica*	Procathepsin L	PF00112.15
FHC00855	2,00E-69	ABU62925	*F. hepatica*	Cathepsin B	PF00112.15
FHC00201	1,00E-112	CAC85636	*F. hepatica*	Legumain like precursor	PF01650.10
FHC00383	2,00E-44	CAC85636	*F. hepatica*	Legumain like precursor	PF01650.10
FHC00251	1,00E-27	CAC85636	*F. hepatica*	Legumain like precursor	-
FHC00413	2,00E-32	CAC85636	*F. hepatica*	Legumain like precursor	-
FHC00456	5,00E-22	Smp002150|29044	*S. mansoni*	Serine protease	PF00089.18
FHC00410	7,00E-09	CPRT0000007748	*S. japonicum*	Probable Ufm1-specific protease 2	-
FHC00435	8,00E-46	B7P5Y9_IXOSC	*I. scapularis*	Calcium-dependent cysteine protease	-
**Proteinase Inhibitors**					
FHC00812	2,00E-16	Q06K58_PHLDU	*P. duboscqi*	Endopeptidase inhibitor	PF10208.1
FHC00195	1,00E-57	AAV68752	*F. hepatica*	cystatin	-
FHC00724	7,00E-13	AAV68752	*F. hepatica*	cystatin	-
**Antioxidant proteins**					
FHC00138	1,00E-131	ACI04165	*F. hepatica*	Thioredoxin peroxidase	PF00578.13
FHC00167	5,00E-37	DQ821492	*Haliotis discus*	Cu/Zn-superoxide dismutase	PF00080.12
FHC00152	9,00E-10	CPRT0000000157	*S. japonicum*	Thioredoxin-like protein	PF06110.3
FHC00111	3,00E-39	AI446859	*E. paraensei*	Glutathione S-Transferase	PF02798.12
FHC00287	5,00E-90	AI446859	*E. paraensei*	μ-Glutathione S-Transferase	PF02798.12
FHC00081	2,00E-57	AT007109	*P. westermani*	Glutathione peroxidase	PF00255.11
FHC00066	7,00E-49	AT006971	*C. sinensis*	Thioredoxin-2 mitochondrial	PF00085.12
**Transmembrane proteins**					
FHC00555	5,00E-26	CPRT0000008170	*S. japonicum*	Clathrin coat-associated protein	PF01217.12
FHC00086	6,00E-32	O01372_SCHJA	*S. japonicum*	22.6 kDa membrane-associated antigen	PF00036.24
FHC00009	9,00E-54	CPRT0000003434	*S. japonicum*	Transmembrane emp24 protein	PF01105.16
FHC00592	2,00E-13	Fgf	*H. sapiens*	FGF receptor activating proteín	PF10277.1
FHC00037	7,00E-34	AAA31753	*F. hepatica*	NADH dehydrogenase subunit 3	PF00507.11
FHC00241	9,00E-53	CPRT0000009505	*S. japonicum*	Succinate dehydrogenase complex, subunit C	PF01127.14
FHC00273	3,00E-24	Smp140000|29115	*S. mansoni*	Tetraspanin-CD63 receptor	PF00335.12
FHC00300	1,00E-11	CPRT0000000388	*S. japonicum*	Ssr4; signal sequence receptor	-
FHC00606	1,00E-06	Smp156020|29231	*S. mansoni*	Glucose transporter	-
**Cell signalling**					
FHC00043	3,00E-31	A4V9Q6_FASHE	*F. hepatica*	Calmodulin-like protein 2	PF00036.24
FHC00494	1,00E-100	CPRT0000000218	*S. japonicum*	Phosphatase 2A inhibitor	PF00956.10
FHC00519	2,00E-10	CED3_CAEEL	*C. elegans*	Caspase-2	PF00656.14
FHC00285	2,00E-43	CPRT0000001178	*S. japonicum*	Cell cycle and apoptosis regulatory protein 1	PF02037.19
FHC00631	1,00E-65	A4IF06_CLOSI	*C. sinensis*	Bax inhibitor factor 1	PF01027.12
FHC00565	3,00E-44	Smp_073560	*S. mansoni*	WD domain G beta-like protein	PF00400.24
FHC00052	7,00E-29	MADD_DROME	*D. melanogaster*	MAP kinase-activating death domain protein	-
**Structural and motor proteins**					
FHC00033	4,00E-74	EL620294	*O. viverrini*	Actin 2	PF00022.11
FHC00117	1,00E-149	EL620294	*O. viverrini*	Actin 2	PF00022.11
FHC00487	1,00E-26	C610909	*L. rubellus*	Actin related protein 2/3	PF04699.6
FHC00379	2,00E-34	EL620325	*O. viverrini*	Cofilin	PF00241.12
FHC00056	7,00E-53	EL620358	*O. viverrini*	Dynein light chain	PF01221.10
FHC00197	1,00E-25	EL619926	*O. viverrini*	Dynein Light Chain	PF01221.10
FHC00363	3,00E-11	EL618949	*O. viverrini*	Dynein LC6	-
FHC00802	4,00E-37	CPRT0000002575	*S. japonicum*	Paramyosin	PF01576.11
FHC00440	1,00E-99	CAP72051	*F. hepatica*	Tubulin beta-3	PF03953.9
FHC00278	7,00E-21	CAP72050	*F. hepatica*	Tubulin beta-2	-

Sequences encoding detoxifying enzymes like thireodoxin peroxidase (TPx), superoxide dismutase (SOD), thioredoxin 2, glutation S-transferases and a novel glutathione peroxidase not previously reported in *F. hepatica *were also found in juveniles, stressing their relevance for immune evasion [72]. In flatworms thioredoxin and glutathione peroxidases are the main enzymes involved in detoxifying reactive oxygen species produced by host immune effector cells [73,74].

Secreted and surface proteins that may modulate host interactions are considered as relevant targets for vaccine or anti-parasitic drug design [75]. SignalP analysis identified putative signal peptides in 60 NEJ predicted proteins, while 52 had an N-terminal signal anchor peptide. Several putative secreted proteins were novel (with no significant hits) or conserved only in trematodes but not detected in other taxa. Some of these transcripts were among the more represented ESTs in juveniles (Table 2). The repeated detection of these transcripts in partial datasets from diverse trematodes support the notion that they are truly highly expressed genes in trematodes, and may be important mediators for parasitism. We selected Contig FHC00023, a predicted secreted protein of unknown function that is the most frequent in the juvenile ESTs with no homologies outside *F. hepatica *for further analysis. By real time PCR we found that this transcript is predominantly expressed in the invasive stage confirming the *in silico *observation (Additional File 9 panel C). The putative ORF is characterized by repeated Ser and Thr residues predicted to be glycosilated, and in further analysis showed faint homology with mucins. Parasite-specific proteins (with no counterparts in vertebrates) like these are ideal targets for development of therapeutic agents since they would have no cross-reactivity with host molecules. The elucidation of the function of these proteins is an important task. The growing availability of functional genomics tools like RNA interference in *F. hepatica *and model trematodes [76-78] offers some hope this can be accomplished.

## Conclusions

The data presented here provides an initial picture of the transcriptional status of the invasive stage of the zoonotic trematode *F. hepatica*, one of the most common parasites of livestock worldwide, and a relevant agent of human disease in impoverished areas of South America and Asia. Besides confirming previously identified genes involved in the invasion process, we also identified plausible candidates for anti-helminthic intervention. A set of putative *F. hepatica *specific transcripts, together with other flatworm specific sequences identified, and a group of transcripts absent in their mammalian hosts, provide an initial framework to pinpoint novel targets for future anti-parasitic drugs or vaccine development. The availability of recently developed functional genomic tools in liver fluke offers a platform to start unraveling the function of these novel conserved genes. Furthermore, we detected interesting differences between the models *Schistosoma *species with other lineages of trematodes, suggesting that genomic and transcriptomic efforts in other flukes might be justified. Comparative studies between diverse trematodes would provide more clues on evolutionary adaptations to parasitism. The richness of information obtained from a limited set of data warrants an in dept analysis of the transcriptome using new multiparallel sequencing technologies.

## Methods

### Parasites

*Fasciola hepatica *metacercariae were obtained in our laboratory from experimentally infected *Lymnaea viatrix *snails and maintained encysted on 0.4% carboxymethyl cellulose until use. Excystment of metacercariae was performed as described previously [15]. Briefly, metacercariae were placed in a 100-μm filter and incubated 5 min with 1% sodium hypochlorite, washed exhaustively with PBS and incubated at 39°C for up to 3 hours in a solution prepared by mixing equal volumes of A (0.4% sodium taurocholate, 120 mM NaHCO_3_, 140 mM NaCl, pH 8.0 and B (50 mM HCl, 33 mM L-cysteine). The emerging NEJs were collected in a 20 μm-filter with RPMI-1640 medium and used for RNA extraction.

### RNA extraction, ligation of RNA adaptors and cDNA synthesis

Total RNA from 1200 NEJs was prepared using the Micro to Midi RNA Extraction Kit (Invitrogen), according to the manufacturer's protocol. Two hundred nanograms of total RNA were used for cDNA synthesis using the protocol described [28]. Briefly, the non capped RNAs were dephosphorylated, and the complete mRNAs were decapped by a pyrophosphatase treatment, and later ligated to the GeneRacer RNA oligo to introduce a 5' priming site in complete mRNAs. After this treatment, first strand synthesis was performed with the reverse transcriptase Superscript III (Invitrogen) using GeneRacer oligo-dT primer (5'GCTGTCAACGATACGCTAC GTAACGGCATGACAGTG(T)_18_3').

### Preparation of NEJ cDNA libraries

Amplification of full-length cDNAs was performed by PCR using universal forward (GeneRacer 5'Nested: 5'GGACACTGACATGGACTGAAGGAGTA3') and reverse primers (GenerRacer 3'Nested: 5'CGCTACGTAACGGCATGACAGTG3') provided by the GeneRacer kit. PCR was carried out for 30 cycles (94°C, 45 sec; 68°C 45 sec; 72°C 5 min) using Hot Start *Taq *DNA polymerase (Fermentas). PCR products were size fractionated in three subpopulations (300-800 bp, 800-2000 bp and >2000 bp) by excision from 1% TBE agarose gels, purified with QIAquick Gel Extraction kit (QIAGEN), ligated to pCR4-TA cloning vectors (Invitrogen), electroporated into One Shot TOP 10 Electrocompetent *E. coli *(Invitrogen), and plated on LB Amp/X-Gal. Recombinant clones from the libraries were randomly picked, grown in Circle Growth medium and stocked at -80°C in 96-well plates in 30% glycerol.

### DNA sequencing and Bioinformatics analysis

Clones were cultured in 96 well plates with Circle Growth media and plasmid DNA was purified by alkaline lysis in 96 well plates. DNA was sequenced with M13 reverse primers using the Dyenamic ET Dye Terminator cycle sequencing kit for MegaBace DNA Analysis Systems (GE Healthcare Life Sciences) according to the manufacturer's instructions.

The sequence reads obtained were processed and analyzed using the Partigene pipeline [29]. Briefly, Trace2dbest [79] processed the chromatograms removing low quality (Phred <15, <150 bp) and vector sequences, and the resulting preprocessed ESTs were assembled in a two-step process carried out by CLOBB [80] and Phrap programs; the resulting contigs and singletons were compared to a set of databases maintained locally (listed in Supplementary Table 1) using tBLASTX and BLASTX. Functional categories were analyzed using annot8r [81]. Signal sequence prediction was performed using SignalP3.0 program [82]. Prediction of trans-membrane domains were conducted using TMHMM software [83]. Blast results comparisons were performed with Simitri [31]. All the available ESTs reads from adult stage of *F. hepatica *available at the Wellcome Trust Sanger Institute http://www.sanger.ac.uk/Projects/Helminths were downloaded and processed with the same pipeline. The juvenile sequences here obtained were deposited at the dbEST with the accessions GT740211 to GT741887.

### Codon usage and amino acid frequencies

For *F. hepatica *adult and juvenile stages, *Echinostoma paraensei*, *Opistorchis viverrini *and *Clonorchis sinensis*, open reading frames were predicted from the assembled EST data using *EMBOSS *bioinformatics suite [84]. The longest ORF from each EST was retrieved and the predicted protein sequence was blasted against the NCBI nr databank. ORFs with significant hits were kept for further analysis. For *S. japonicum *and *S. mansoni *predicted coding regions obtained through the respective genome projects were analyzed. Codon and amino acid usage was calculated using GCUA: General Codon Usage Analysis tool [85]

### Real time PCR

Real time PCR experiments were carried out in an Applied Biosystems 7500 Real time PCR System. Ten microliters of different dilutions of cDNA of NEJ and adult parasites were amplified using 0.2 μM each specific primers, 1.5 mM MgCl_2_, 25 uM dNTPs, 0.25 U Platinun *Taq *DNA polymerase (Invitrogen), 1× SYBR Green, 1× PCR buffer in a 20 uL volume reaction. Primers sequences are β-actin (Forward 5'-GTGTTGGATTCTGGTGATGGTGTC-3' and Reverse 5'-CAATTTCTCCTTGAT GTCTCG-3'), FHC00023 (Forward 5'-ATGGTGCGAACGCTAAG-3' and Reverse 5'-GAAGAACGCAACGCCGAAGA-3'), Legumain 1 (Forward 5'-CAAGGATGTTTATGAAGGG-3'and Reverse 5'-TGCTTTGTTCATGCTGGC-3') Legumain 3 (Forward 5'-AGCAGACAAAACCCTTATCGT-3'and Reverse 5'-GGAATAATAGTAGGCGACGTG-3'). Reactions were performed in triplicate using the following PCR amplification conditions, 1 cycle (94°C, 5 min), 40 cycles (94°C, 15 seg; 60°C, 10 seg; 72°C, 15 seg). All results were analyzed using the 2^-ΔΔCt ^method and β-actin as internal control group [86].

### Note added in proof

Recently a separate study describing the generation of more than 500,000 sequences from an adult cDNA library using 454 sequencing was published [87]. However, at the time of writing these sequences have not been made publically accessible and hence a comparative analysis of this dataset was not possible.

## Authors' contributions

MC, CC, AZ, & JFT conceived the work. MC, GR, LR & NDO obtained the RNA and generated the libraries. MC, ES, NR & NDO amplified the library and sequenced the clones. NR, PS & FAV organized the analysis pipeline, and MC, NR, NDO & PS processed the sequence data, MC, CC, AZ and JFT wrote the manuscript, which was discussed, improved and corrected by all participant authors. All authors read and approved the final manuscript.

## Supplementary Material

Additional file 1 **Figure S1. cDNA library generation procedure**. **(A) **Diagrammatic representation of the major steps in the cDNA library construction. **(B) **Details on the full length selection and adapter ligation. **(C) **Representative PCR products from colonies obtained after size fractionation; left: small size insert library, right: large size insert library. Click here for file

Additional file 2 **Table S1- Databases used in this study**. Details and links to the databases used in this study. Click here for file

Additional file 3 **Table S2- Overview of *F. hepatica *adult ESTs assembly**. Details of the assembly of the available adult stage ESTs with the Partigene pipeline. Click here for file

Additional file 4 **Figure S2. Three way comparisons of *F. hepatica *juvenile contigs against early metazoans and model organisms**. **(A) **The complete set of contigs generated by the Partigene compared to ESTs from the early metazoans (non bilaterians) *Trichoplax adherens*, Porifera (sponges) and Cnidaria (jellyfish and corals). **(B) **Comparison among the nematode *C. elegans*, the insect *D. melanogaster *and the arachnid (thick) *I. scapularis*. **(C) **Comparison to the vertebrates *D. renio *(zebra fish), *G. gallus *(chicken) and *H. sapiens *(human). Click here for file

Additional file 5 **Table S3- Ribosomal proteins detected in NEJ EST assembly**. List of ribosomal proteins detected in the juvenile assembly. Click here for file

Additional file 6 **Table S4- Most abundant contigs in the *F. hepatica *adult EST assembly **Details of the contigs containing more reads in the adult stage assembly. Click here for file

Additional file 7 **Figure S3. Phylogenetic tree of Fasciolidae cathepsins B**. Bootstrapped neighbor joining tree of available cathepsin B coding sequences, showing the clustering of juvenile and adult forms. Sequences are color coded by their stage origin: adult stage represented in red rhombs, juveniles in blue triangles and metacercariae in green circles. Contig sequences from ESTs projects (Sanger Center and this study) are unfilled. Sequences from *F. gigantica *are underlined. Sequences from GeneBank are named following the same criterion of Robinson et al [14], namely the first two characters indicate species (Fh or Fg for *F. hepatica *or *F. gigantica *respectively) followed by the cathepsin type, country of origin, accession, stage and P or C for describing partial or complete coding sequences respectively. The "adult" and "juvenile" clusters observed are not due to sample bias since they are maintained when analyzing partial regions corresponding to 5' or 3'ends of the ESTs (data not shown). The nucleotide sequence alignment of the cathepsins B used to generate the tree is available as Additional File 8. Click here for file

Additional file 8 **Supplementary Data S1 - Alignment of cathepsin B sequences**. Nucleotide sequence alignment of cathepsin B sequences. Click here for file

Additional file 9 **Figure S4. Differentially expressed genes in *F. hepatica***. Transcriptional levels of legumain 1 **(A) **legumain 3 **(B) **and Contig FHC00023 **(C) **were determined by Real time RT-PCR in newly excysted juveniles and adults. Levels were measured by the 2-delta delta CT method using actin as a control for normalization. Click here for file
