# Supplementary figures and images for: Survey of transcripts expressed by the invasive juvenile stage of the liver fluke Fasciola hepatica
Source: BMC Genomics. 2010 Apr 7;11:227. doi: 10.1186/1471-2164-11-227 (PMC2867827; doi:10.1186/1471-2164-11-227)

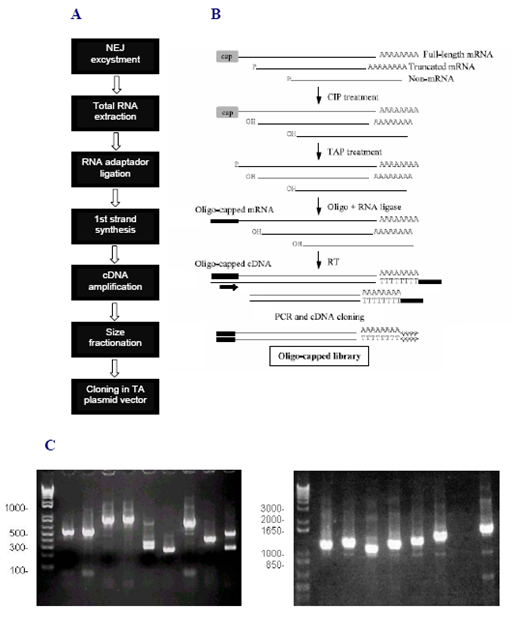

Supplement: Additional file 1 — Figure S1. cDNA library generation procedure. (A) Diagrammatic representation of the major steps in the cDNA library construction. (B) Details on the full length selection and adapter ligation. (C) Representative PCR products from colonies obtained after size fractionation; left: small size insert library, right: large size insert library. [file 1471-2164-11-227-S1.TIFF]

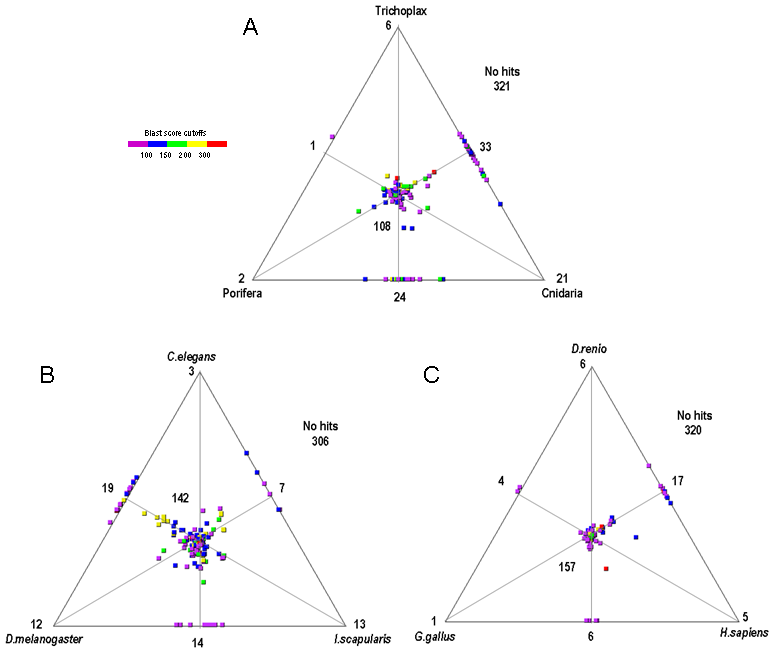

Supplement: Additional file 4 — Figure S2. Three way comparisons of F. hepatica juvenile contigs against early metazoans and model organisms. (A) The complete set of contigs generated by the Partigene compared to ESTs from the early metazoans (non bilaterians) Trichoplax adherens, Porifera (sponges) and Cnidaria (jellyfish and corals). (B) Comparison among the nematode C. elegans, the insect D. melanogaster and the arachnid (thick) I. scapularis. (C) Comparison to the vertebrates D. renio (zebra fish), G. gallus (chicken) and H. sapiens (human). [file 1471-2164-11-227-S4.TIFF]

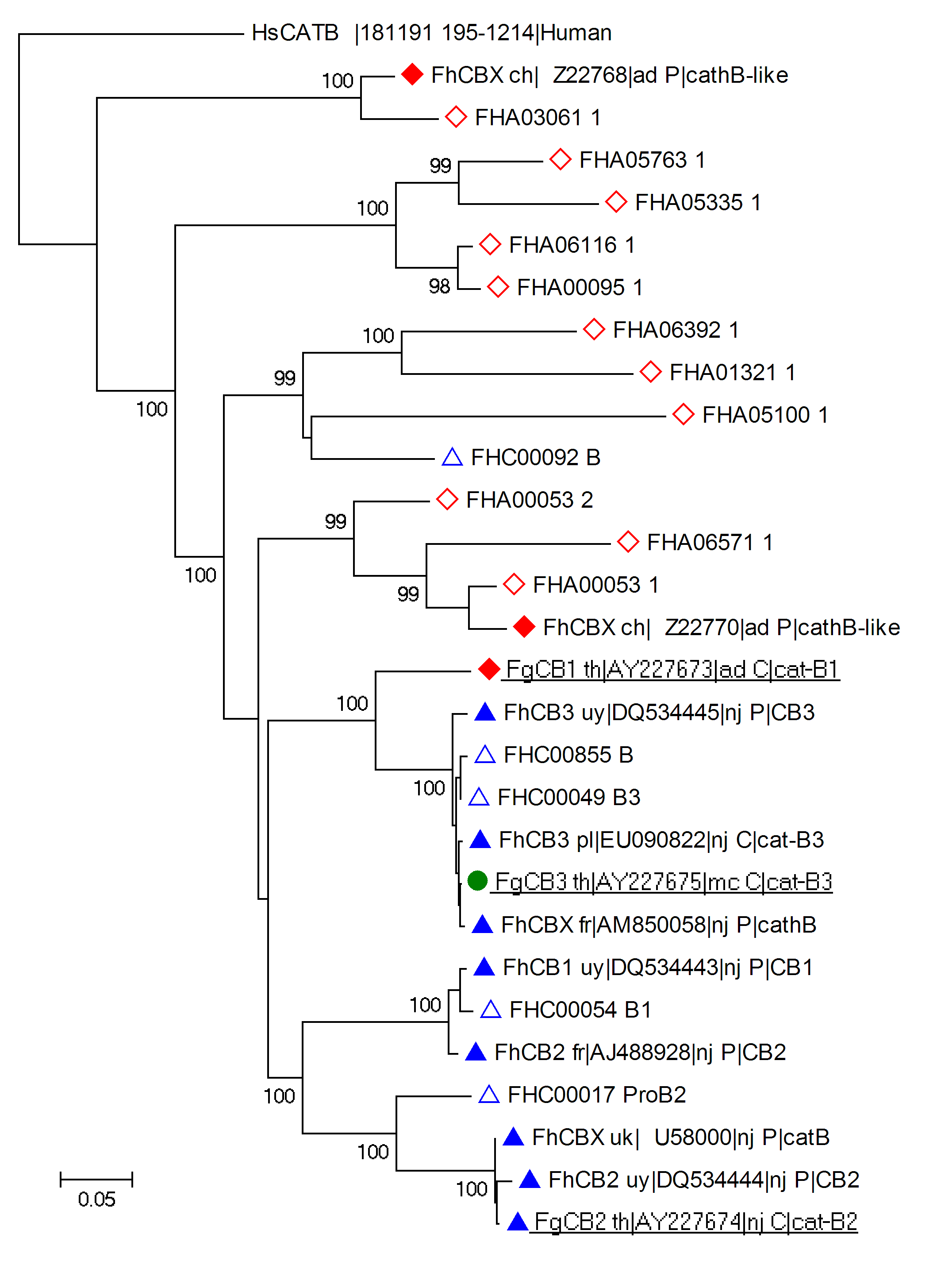

Supplement: Additional file 7 — Figure S3. Phylogenetic tree of Fasciolidae cathepsins B. Bootstrapped neighbor joining tree of available cathepsin B coding sequences, showing the clustering of juvenile and adult forms. Sequences are color coded by their stage origin: adult stage represented in red rhombs, juveniles in blue triangles and metacercariae in green circles. Contig sequences from ESTs projects (Sanger Center and this study) are unfilled. Sequences from F. gigantica are underlined. Sequences from GeneBank are named following the same criterion of Robinson et al [14], namely the first two characters indicate species (Fh or Fg for F. hepatica or F. gigantica respectively) followed by the cathepsin type, country of origin, accession, stage and P or C for describing partial or complete coding sequences respectively. The "adult" and "juvenile" clusters observed are not due to sample bias since they are maintained when analyzing partial regions corresponding to 5' or 3'ends of the ESTs (data not shown). The nucleotide sequence alignment of the cathepsins B used to generate the tree is available as Additional File 8. [file 1471-2164-11-227-S7.TIFF]

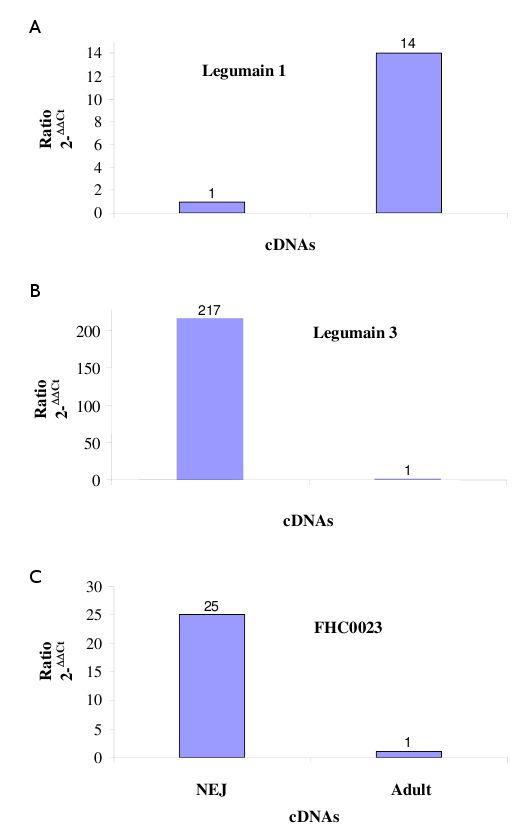

Supplement: Additional file 9 — Figure S4. Differentially expressed genes in F. hepatica. Transcriptional levels of legumain 1 (A) legumain 3 (B) and Contig FHC00023 (C) were determined by Real time RT-PCR in newly excysted juveniles and adults. Levels were measured by the 2-delta delta CT method using actin as a control for normalization. [file 1471-2164-11-227-S9.TIFF]
