# Supplementary material for: Survey of transcripts expressed by the invasive juvenile stage of the liver fluke Fasciola hepatica
Source: BMC Genomics. 2010 Apr 7;11:227. doi: 10.1186/1471-2164-11-227 (PMC2867827; doi:10.1186/1471-2164-11-227)
Supplement: Additional file 2 — Table S1- Databases used in this study. Details and links to the databases used in this study. [file 1471-2164-11-227-S2.PDF]

Additional File 2- Table S1- Databases used in this study

Taxonomic group	Species	Sequences		Type	Source
		Raw seqs	Processed		
Basal	*Monsiga brevicollis*		9196	mRNA models	JGI [1]
metazoans	*Trichoplax adherens*		11520	mRNA models	JGI
Porifera	*Various*	17466		ESTs	dbEST[2]
Cnidaria	*Hydra magnipapillata*		12761	Unigene models	Unigene[3]
	Nematostella vectensis		19167	Unigene models	Unigene
	Nematostella vectensis		27273	mRNA models	JGI
Trematoda	*Fasciola hepatica*	10413	3646	Contigs	WTSC[4]
	Echinostoma paraensei	358		ESTs	dbEST
	Clonorchis sinensis	2970		ESTs	dbEST
	Opistorchis viverrini	4194		ESTs	dbEST
	Paragonimus westermani	505		ESTs	dbEST
	Schistosoma mansoni		13293	mRNA models	GENEDB[5]
	Schistosoma japonicum		12657	mRNA models	SGST[6]
Cestoda	*Echinococcus granulosus*	9701		ESTs	dbEST
	Echinococcus multilocularis	1168		ESTs	dbEST
	Mesocestoides cortii	1783		ESTs	dbEST
	Monieza expansa	1098		ESTs	dbEST
	Taenia solium	24450		ESTs	dbEST
Turbellaria	*Convoluta pulchra*	2128		ESTs	dbEST
	Dugesia japonica	7362		ESTs	dbEST
	Dugesia ryukyuensis	8988		ESTs	dbEST
	Macrostomum lignano	7617		ESTs	dbEST
	Schmidtea mediterranea	75029		ESTs	dbEST
	Symsagittifera roscoffensis	846		ESTs	dbEST
Mollusca	*Lottia gigantea*		15602	mRNA models	Unigene
	Aplysia californica		23079	mRNA models	Unigene
	Haliotis discus	2028		ESTs	dbEST
	Euprymna scolopes	35420		ESTs	dbEST
	Biomphalaria glabrata	52624		ESTs	dbEST
	Lymnaea stagnalis	1320		ESTs	dbEST
Annelida	*Hirudo medicinalis*	26833		ESTs	dbEST
	Lumbricus rubellus	19934		ESTs	dbEST
	Helobdella robusta		23432	mRNA models	JGI
	Capitella spp.		32415	mRNA models	JGI
Nematoda	*Caenorhabditis elegans*		29603	mRNA models	Ensembl[7]
Insecta	*Drosophila melanogaster*		20909	mRNA models	Ensembl
Arachnida	*Ixodes scapularis*		20486	mRNA models	Ensembl
Chordata	*Danio renio*		28717	mRNA models	Ensembl
	Gallus gallus		22290	mRNA models	Ensembl
	Homo sapiens		54617	mRNA models	Ensembl

Links
[1] /genome.jgi-psf.org/,
[2] /www.ncbi.nlm.nih.gov/dbEST/
[3] /www.ncbi.nlm.nih.gov/unigene/
[4] /www.sanger.ac.uk/Projects/Helminths/
[5] /www.genedb.org/
[6] /lifecenter.sgst.cn/
[7] /www.ensembl.org/
