# Supplementary material for: Survey of transcripts expressed by the invasive juvenile stage of the liver fluke Fasciola hepatica
Source: BMC Genomics. 2010 Apr 7;11:227. doi: 10.1186/1471-2164-11-227 (PMC2867827; doi:10.1186/1471-2164-11-227)
Supplement: Additional file 3 — Table S2- Overview of F. hepatica adult ESTs assembly. Details of the assembly of the available adult stage ESTs with the Partigene pipeline. [file 1471-2164-11-227-S3.PDF]

Additional File 3- Table S2- Overview of *F. hepatica* adult ESTs assembly

ADULT LIBRARY	Partigene
EST analyzed	10753
Submissable EST	10588
Contigs	4089
Clusters	1879
Singletons	2210
Contigs with Blast hits	2674
Contigs with GO assignments	ND
Contigs with Pfam hits	ND
Average insert size	ND

ND: not determined
