# Supplementary material for: Survey of transcripts expressed by the invasive juvenile stage of the liver fluke Fasciola hepatica
Source: BMC Genomics. 2010 Apr 7;11:227. doi: 10.1186/1471-2164-11-227 (PMC2867827; doi:10.1186/1471-2164-11-227)
Supplement: Additional file 5 — Table S3- Ribosomal proteins detected in NEJ EST assembly. List of ribosomal proteins detected in the juvenile assembly. [file 1471-2164-11-227-S5.PDF]

Additional File 5 - Table S3- Ribosomal proteins detected in NEJ EST assembly

seq id	Seq start	seq end	hmm acc	hmm start	hmm end	Evalue	HMM name and Description
fhc00425_1	22	63	PF00428.11	1	41	1.3e-06	60s Acidic ribosomal protein
fhc00359_1	47	79	PF01929.9	1	33	7.9e-09	Ribosomal protein L14
fhc00155	13	167	PF00252.10	1	150	7.8e-57	Ribosomal protein L16p/L10e
fhc00343_1	5	175	PF01775.9	1	175	4.7e-106	Ribosomal L18ae protein family"
fhc00602_1	10	43	PF00828.11	1	35	1.1e-17	Eukaryotic ribosomal protein L18
fhc00028_1	26	162	PF00861.14	1	145	1.9e-54	Ribosomal L18p/L5e family
fhc00157_1	96	231	PF03947.10	1	136	9.6e-60	Ribosomal Proteins L2, C-terminal domain
fhc00087_1	18	135	PF01776.9	1	115	7.1e-24	Ribosomal L22e protein family
fhc00216_1	22	101	PF00276.12	1	96	6.9e-30	Ribosomal protein L23
fhc00069_1	2	104	PF01778.9	1	134	1.5e-18	Ribosomal L28e protein family
fhc00313	6	66	PF00831.15	1	59	4.9e-12	Ribosomal L29 protein
fhc00213_1	1	51	PF00327.12	1	54	5.6e-23	Ribosomal protein L30p/L7e
fhc00199_1	12	106	PF01198.11	1	97	1,0e-54	Ribosomal protein L31e
fhc00709_1	1	44	PF01655.10	69	115	9.5e-17	Ribosomal protein L32
fhc00317_1	1	90	PF01199.10	1	101	3.9e-46	Ribosomal protein L34e
fhc00222_1	4	102	PF01158.10	1	109	5.3e-30	Ribosomal protein L36e
fhc00306_1	1	80	PF01780.11	1	90	1.4e-47	Ribosomal L37ae protein family
fhc00074_1	2	55	PF01907.11	1	55	2.3e-34	Ribosomal protein L37e
fhc00819_1	2	69	PF01781.10	1	77	1.3e-34	Ribosomal L38e protein family
fhc00144_1	1	65	PF00935.11	1	80	4.6e-32	Ribosomal protein L44
fhc00324_1	63	162	PF00673.13	1	109	3.3e-55	Ribosomal L5P family C-terminus
fhc00360	124	161	PF01159.11	1	40	3.6e-18	Ribosomal protein L6e
fhc00448_1	1	51	PF01248.18	45	101	2.5e-13	Ribosomal protein L7Ae/L30e/S12e/Gadd45 family
fhc00457_1	17	113	PF00338.14	1	101	1.5e-32	Ribosomal protein S10p/S20e
fhc00609_1	12	146	PF00164.17	1	141	1.4e-53	Ribosomal protein S12
fhc00057_1	17	143	PF00416.14	1	131	6.2e-69	Ribosomal protein S13/S18
fhc00128_1	1	60	PF08069.4	1	60	3.2e-37	Ribosomal S13/S15 N-terminal domain
fhc00013_1	3	56	PF00253.13	1	57	3.3e-21	Ribosomal protein S14p/S29e
fhc00454_1	61	131	PF00366.12	1	71	8.1e-36	Ribosomal protein S17
fhc00281_1	29	110	PF00203.13	1	82	3.3e-40	Ribosomal protein S19
fhc00175_1	18	186	PF00318.12	1	216	6.1e-76	Ribosomal protein S2
fhc00136_1	1	82	PF01249.10	1	83	2.6e-42	Ribosomal protein S21e
fhc00062_1	7	86	PF01282.11	1	84	1,0e-32	Ribosomal protein S24e
fhc00016_1	1	74	PF03297.7	1	111	2.5e-34	Ribosomal protein S25
fhc00356_1	5	119	PF01283.11	1	114	4.1e-51	Ribosomal protein S26e
fhc00146_1	28	82	PF01667.9	1	55	3.3e-27	Ribosomal protein S27
fhc00137_1	1	54	PF01200.10	1	71	1.8e-20	Ribosomal protein S28e
fhc00305_1	1	54	PF01200.10	1	71	1.8e-20	Ribosomal protein S28e
fhc00255_1	106	197	PF00189.12	1	89	1.8e-24	Ribosomal protein S3, C-terminal domain
fhc00027_1	1	203	PF01015.10	1	215	7.6e-93	Ribosomal S3Ae family
fhc00298_1	5	106	PF00163.11	1	110	3.2e-39	ibosomal protein S4/S9 N-terminal domain
fhc00813_1	1	75	PF00900.12	1	85	2.2e-39	Ribosomal family S4e
fhc00029_1	2	75	PF03719.7	1	75	3.9e-35	Ribosomal protein S5, C-terminal domain
fhc00712_1	1	187	PF01251.10	1	190	4.9e-79	Ribosomal protein S7e
fhc00517_1	1	50	PF01201.14	244	289	3.5e-21	Ribosomal protein S8e
fhc00105	7	139	PF00380.11	1	138	1.5e-70	Ribosomal protein S9/S16
