# Supplementary material for: Survey of transcripts expressed by the invasive juvenile stage of the liver fluke Fasciola hepatica
Source: BMC Genomics. 2010 Apr 7;11:227. doi: 10.1186/1471-2164-11-227 (PMC2867827; doi:10.1186/1471-2164-11-227)
Supplement: Additional file 6 — Table S4- Most abundant contigs in the F. hepatica adult EST assembly Details of the contigs containing more reads in the adult stage assembly. [file 1471-2164-11-227-S6.PDF]

Additional File 6- Table S3 - Most abundant contigs in the *F. hepatica* adult EST assembly

Contigs	Reads	Eval	Uniprot Hit	Description	Cathepsin classification *
FHA00118	216	7E-94	CATLL_FASHE	Cathepsin L-like proteinase precursor - Fasciola hepatica (Liver fluke)	FHCL1A_au3
FHA00235	206	0	Q24944_FASHE	Cathepsin L-like protease Fasciola hepatica	FHCL2__chC
FHA00254	118	8E-33	A7XWR4_CLOSI	ADP/ATP carrier (Fragment) Clonorchis sinensis	
FHA00014	93	1E-36	Q0ZNJ0_FASGI	Secreted saposin-like protein SAP-3 Fasciola gigantica	
FHA00676	84	1E-102	CATLL_FASHE	Cathepsin L-like proteinase precursor - Fasciola hepatica (Liver fluke)	FHCL1A_au3
FHA00010	83	1E-178	Q7JNQ9_FASHE	Secreted cathepsin L 1 Fasciola hepatica CL1	FHCL1A_ie1
FHA00002	79	1E-130	ALF_ECHMU	Fructose-bisphosphate aldolase - Echinococcus multilocularis	
FHA00026	68	2E-41	A9XXK5_MASGI	Putative vacuolar ATP synthase subunit B (Fragment) Mastigoproctus giganteus	
FHA00030	58	0	CATLL_FASHE	Cathepsin L-like proteinase precursor - Fasciola hepatica (Liver fluke)	FHCL1A_au3
FHA00067	52	4E-38	B5G4X7_CLOSI	Lysosomal pro-X carboxypeptidase (Fragment) Clonorchis sinensis	
FHA00086	42	5E-52	B6ZBP3_SCHMA	Saposin-like protein Schistosoma mansoni SAP	
FHA00238	37	4E-33	Q5DC83_SCHJA	SJCHGC00439 protein Schistosoma japonicum	
FHA01545	36	2E-12	Q5D9H4_SCHJA	SJCHGC00969 protein Schistosoma japonicum	
FHA01165	35	1E-115	CATLL_FASHE	Cathepsin L-like proteinase precursor - Fasciola hepatica (Liver fluke)	FHCL1A_au3
FHA00085	32	5E-42	Q5DGI2_SCHJA	SJCHGC06317 protein Schistosoma japonicum	
FHA00051	31	0	Q9NB30_FASHE	Cathepsin L Fasciola hepatica	FHCL5__au5
FHA00009	31			No Significant Hit	
FHA00117	28			No Significant Hit	
FHA00006	27	1E-07	B6NYV7_BRAFL	Putative uncharacterized protein Branchiostoma floridae BRAFLDRAFT_129456	
FHA00105	27	1E-126	Q7ZYU9_XENLA	Alphatubulin 84b-prov protein Xenopus laevis alphatub84b-prov	
FHA00439	26	1E-115	Q6R018_FASHE	Cathepsin L protein Fasciola hepatica	FHCL1A_pt1
FHA00356	26	1E-151	A5Z1V3_FASHE	Secreted cathepsin L2 Fasciola hepatica CL2	FhcL2__tr
FHA00386	25	1E-134	Q3UJU1_MOUSE	Putative uncharacterized protein Mus musculus Mut	
FHA00095	25	1E-113	Q8MNY2_SCHMA	Cathepsin B1 isotype 1 Schistosoma mansoni cb1.1	FhCB1_us
FHA00908	25	8E-38	Q5D2M7_9TREM	Myoglobin 1 Paragonimus westermani myo1	
FHA00920	25	2E-72	Q5DFM2_SCHJA	SJCHGC01661 protein Schistosoma japonicum	

Cathepsin classification according to Robinson et al. [14]
